# Case report of a laryngotracheal reconstruction with anterior and posterior costal cartilage graft and stent placement – Surgical technique

**DOI:** 10.1016/j.ijscr.2019.04.009

**Published:** 2019-04-18

**Authors:** Nathan Montoya Albrecht, Samuel Ostrower

**Affiliations:** a Edward Via College of Osteopathic Medicine, Auburn, AL, United States; b Pediatric Otolaryngology Joe DiMaggio Children’s Hospital, Hollywood, FL, United States

**Keywords:** Case report, Grade III subglottic stenosis, Grade IV subglottic stenosis, Anterior costal cartilage graft, Posterior costal cartilage graft

## Abstract

**Objective:**

The purpose of this report is to examine a grade III subglottic stenosis case with double-stage laryngotracheal reconstruction using a costal cartilage graft and to explore the relevant literature regarding these topics.

**Context:**

In this report, the authors examine a severe grade III subglottic stenosis pediatric case and its subsequent laryngotracheal reconstruction with costal cartilage graft. This case demonstrates a surgical intervention which has become infrequently utilized since neonatologists began using noninvasive positive pressure airway support rather than intubation; thus, the incidence of acquired subglottic stenosis is decreasing. Features of the case and its surgical correction are discussed in detail.

**Case Report Summary:**

The patient is a 3-year-old African-American female born prematurely at 30 weeks by emergent cesarean section complicated by placenta previa, twin gestation and absent end diastolic flow. The patient has a past medical history significant for apnea of prematurity, necrotizing enterocolitis, bronchopulmonary dysplasia, hyaline membrane disease, pneumonia, abnormal electroencephalogram, cardiac arrest due to respiratory disorder, parainfluenza infection, chronic respiratory failure, laryngomalacia and grade III subglottic stenosis. The patient’s surgical history is significant for supraglottic laryngoplasty, tracheostomy placement, gastrostomy tube placement and laryngotracheal reconstruction with anterior and posterior costal cartilage graft and stent placement with subsequent stent removal. As of March 2019, the patient continues to have follow up laryngoscopy/bronchoscopy with dilation. Ultimately, the patient will maintain her own airway with tracheostomy tube removal.

**Data sources and overall comparisons:**

A PubMed literature review of subglottic stenosis and laryngotracheal reconstruction with costal cartilage graft was performed. Twenty sources were reviewed leading to greater understanding of the evidence supporting the laryngotracheal reconstruction with graft treatment modality.

**Conclusion:**

Optimal management and correction of subglottic stenosis is possible via laryngotracheal reconstruction with cartilage graft. The reconstruction procedure, first utilized in the 1970’s, allows correction of severe (grades III and IV) subglottic stenosis. After healing is complete, the patient regains function of her own airway.

## Highlights

• Subglottic stenosis is defined as airway narrowing below the true vocal folds.
• Subglottic stenosis commonly occurs after endotracheal intubation in newborns.
• The incidence of subglottic stenosis has decreased to 1–2% of premature patients.
• The single-stage procedure avoids prolonged hospitalization.
• The two-step procedure allows for treatment of a more extensively stenosed area.

## 1 Introduction

Subglottic stenosis is defined as airway narrowing below the true vocal folds. In approximately 5% of cases subglottic stenosis is congenital and in the other 95% of cases it is acquired – typically from prolonged intubation, direct trauma, smoke inhalation or from chronic infections/inflammatory diseases. Subglottic damage resulting in subglottic stenosis commonly occurs after endotracheal intubation in newborns, especially in premature infants with low body weight or in those who require prolonged endotracheal tube ventilation [1]. It is thought that stenosis is due to epithelial damage after endotracheal intubation/artificial airway trauma that leads to necrosis, edema and ulceration, granulation tissue proliferation that results in anatomical dysfunction. Whited has written that days of intubation are directly correlated to percentage of stenosis with 2–5 days of intubation leading to 0–2% stenosis, 5–10 days intubation leading to 4–5% stenosis and more than 10 days of intubation leading to 12–14% stenosis [2]. There is a risk that patients may acquire long-term pulmonary issues (i.e., persistent lobar atelectasis) due to a stenotic airway [3]. Recently, neonatologists have started using noninvasive positive pressure airway support for infants born with chronic lung disease, rather than intubation for these patients. Because of this, severe subglottic stenosis and its correction by open larngotracheal reconstruction is becoming an infrequently seen condition and surgical treatment. According to McClay and Meyers, the incidence of acquired subglottic stenosis after endotracheal intubation has decreased from 24% of premature infant patients requiring intubation in the 1960′s to 1–2% of premature patients in the late 1990's [4].

Most commonly, patients will demonstrate stridor (typically biphasic) within 2–4 weeks post-extubation if they have a moderate or severe degree of subglottic stenosis [3]. These patients require endoscopic evaluation (rigid endoscopy is the gold standard) to determine the severity of subglottic stenotic airway obstruction post-extubation and may require tracheostomy for months or years until reconstructive laryngotracheoplasty can be performed [5,6]. Additionally, patients which subglottic stenosis should be treated empirically with proton pump inhibitors as studies have consistently shown that these patients have a greater incidence of gastroesophageal reflux compared with the overall pediatric population [[7], [8], [9]].

Explanation of a single-stage reconstructive laryngotracheoplasty procedure with cartilage graft was first documented in the medical literature in 1972 when Fearon and Cotton wrote of their experiment with a “one-stage surgical procedure” that avoided prolonged hospitalization by allowing for removal of a tracheotomy tube right after surgical repair [10]. Prior to this study, most laryngotracheal stenosis cases were managed by endoscopic dilatation which was useful for grade I (0–50% narrowing) and grade II (51–70% narrowing) stenosis, but often not robust enough for higher grade stenosis (grade III at 71–99% narrowing and grade IV with complete obstruction with no detectable lumen) [11]. [Note: this is the Cotton-Myer system of subglottic stenosis classification]. In 2006, Herrington, Webber, and Anderson documented that almost 70% of patients who received dilation required repeated follow-up procedures to maintain the gains accomplished by the initial treatment [12]. Since Fearon and Cotton’s 1970′s publication, their single-stage technique has become the standard treatment for laryngotracheal stenosis in the pediatric population [13]. It can produce an adequate airway with competent larynx while preserving, as much as possible, the patient’s own anatomy and voice (Table 1).

**Table 1:** Benign esophageal shwannomas case reports searched in Pubmed database during the last 8 years.

Author	Year	Age	Sex	Location	Depth	Size	Symptoms	Management
Choo et al.	2011	22	M	Upper thoracic esophagus	Submucosa	80 × 60 × 30 mm	Cough, dyspnea and dysphagya	Enucleation
Liu Tieqin et al.	2013	62	F	NA	Submucosa	90 × 40 × 30 mm	Dysphagia and dyspnea	Partial esophagectomy and esophagogastrostomy
Kitada M et al.	2013	55	F	Upper to middle mediastinum	Submucosa	75 × 57 × 80 mm	Palpitations and dysphagia	Mini Thoracotomy
Jeon Hyu Woo et al.	2014	32	F	Upper thoracic esophagus	Submucosa	87 × 59 × 24 mm	Chest pain	Surgical enuclation
Jeon Hyu Woo et al.	2014	63	M	Upper thoracic esophagus	NA	Two lobes: 95 × 70 × 65 mm 88 × 50 × 55 mm	No symptoms	Thoracotomy
Gu et al.	2014	39	M	Upper mid	Submucosa	35 × 32 × 12 mm	Obstructive sensation	Surgical esophagectomy
Tomono et al.	2015	59	F	Middle thoracic esophagus	Submucosa	109 × 7.2 × 7.1 mm	Dysphagia, dyspnea, disturbed consciousness	Subtotal esophagectomy
Wang, et al.	2015	53	F	NA	NA	NA	NA	Surgical excision
Wang, et al	2015	52	F	NA	NA	NA	NA	Surgical excision
Zhang Q, et al.	2016	67	F	NA	NA	NA	Dysphagia, foreign body	Surgical excision
Watanabe, et al.	2016	39	F	Upper third	Submucosa	55 × 45 × 24 mm	Epigastric pain, difficulty swallowing	Surgical excision
Moro K, et al.	2017	66	M	Upper third	Submucosa	52 × 40 × 31 mm	Dysphagia	Surgical excision
Onodera Y, et al.	2017	47	F	Aortic Arch	Submucosa	60 mm	Dysphagia	Thoracoscopic + endoscopic excision
Current article	2018	40	F	Upper third	Submucosa	80 × 45 × 20 mm	Pharyngitis, odynophagia, dysphagia	Surgical excision

NA: Not available.

**Fig. A1:**
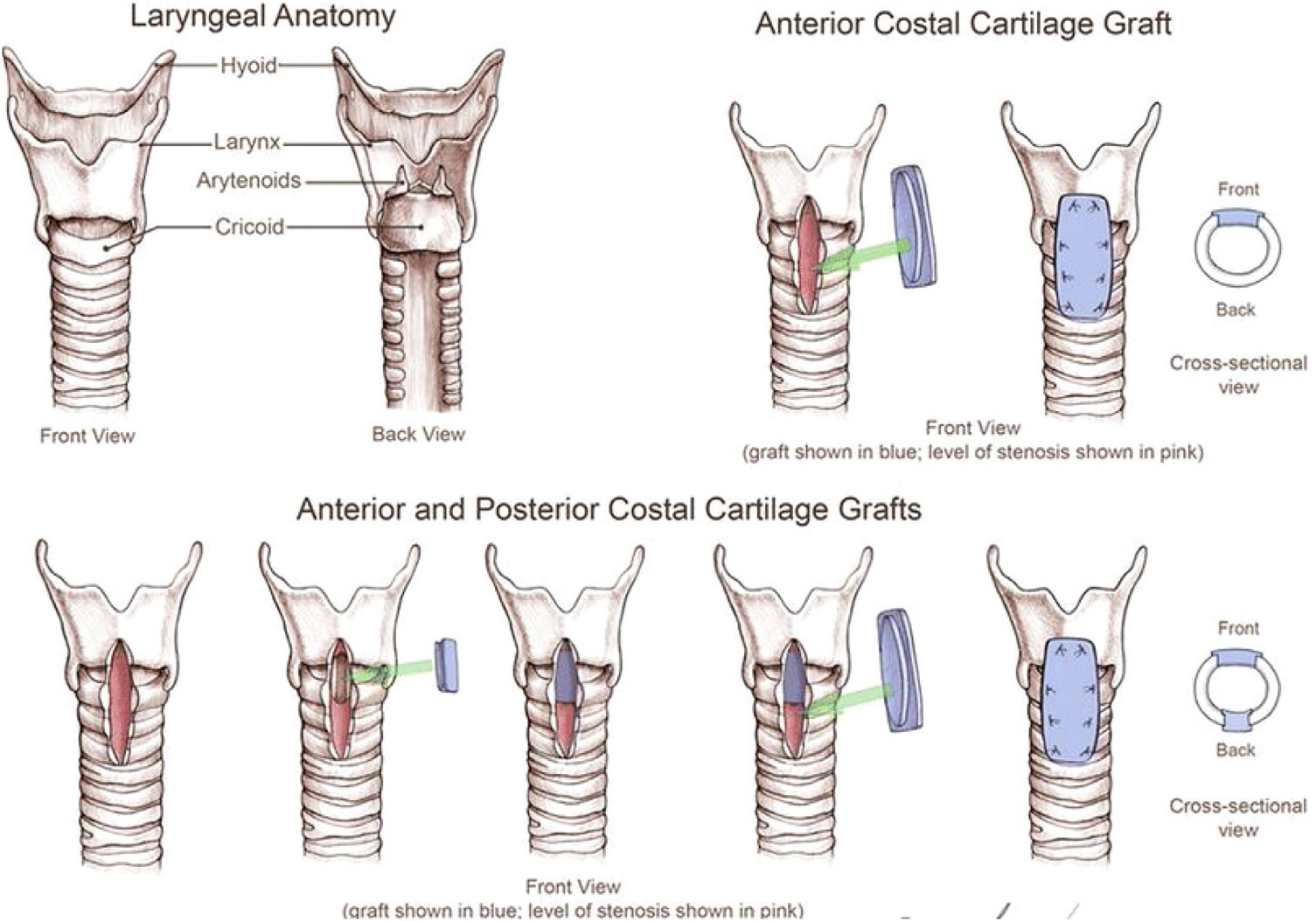
Laryngeal Anatomy, Anterior Costal Cartilage Graft and Anterior and Posterior Costal Cartilage Grafts. Image: Prickett K.K., Jacobs I.N. (2015) Neonatal Subglottic Stenosis. In: Lioy *J*., Sobol S. (eds) Disorders of the Neonatal Airway. Springer, New York, NY.

The single-stage laryngotracheoplasty procedure is a combination of an anterior cricoid and tracheal split with cartilage graft and stenting (Fig. A1). In the procedure, the stenotic laryngotracheal segment is divided along a sagittal line and is expanded and stabilized with an autologous cartilage graft commonly from the auricular, thyroid, or costal cartilage (based on size requirement, with the cartilage graft providing the largest possible size) ([14]). After the procedure the tracheotomy tube can be removed immediately. In contrast, the two-step or double-stage procedure is identical to the single-stage laryngotracheoplasty procedure, except that a posterior incision is also made and grafted. This allows for treatment of a more extensively or circumferentially stenosed area, or for the treatment of a patient with posterior subglottic stenosis; however, it does not allow for immediate removal of the tracheotomy tube. Instead, this correction is typically stented for several weeks before the tracheotomy tube is removed.

The single-stage laryngotracheoplasty has a better overall decannulation rate with fewer post-reconstruction procedures than the double-stage procedure, but the overall decannulation rate is not significantly lower in the double-stage procedure: 86–100% for the single-stage procedure vs. 83–93% for the double-stage procedure [[15], [16], [17]]. The greatest advantage of the two-stage procedure is that it allows for treatment of a more severely stenotic area or the treatment of an area of prior laryngeal surgery [15].

According to a review of two hundred pediatric single-stage laryngotracheal reconstruction surgeries conducted in the year 2000, authors Gustafson et al. state that surgeons must be wary of the following factors which may increase the risk of reintubation after laryngotracheoplasty: using an anterior and posterior costal cartilage graft, if the patient is younger than 4 years old, sedation lasting greater than 48 h, a leak pressure around the endotracheal tube greater than 20 cm H2O, and if there was moderate to severe tracheomalacia pre-procedure [18]. The patient discussed below does share some of the preceding risk factors including the use of an anterior and posterior costal cartilage graft, being less than 4 years old, and did have moderate to severe tracheomalacia pre-procedure. However, the patient was sedated for less than 48 h and there was no leak around the endotracheal tube. The patient did not require reintubation.

In 2006, authors Nouraei et al., reported observing stent colonization with S. *aureus* and P. *aeruginosa* with concomitant increased rates of granulation tissue formation after laryngotracheoplasty. The authors suggested the use of antibiotics with coverage for S. *aureus* and P. *aeruginosa* for one week post-operatively for patients undergoing the procedure [19]. In the case that follows, observe that our patient was found have sputum cultures positive for *P*. *aeruginosa* on post-op day 4 after layngotracheoplasty. The patient was treated appropriately.

In 2007, a 10-year retrospective study examined over 70 pediatric laryngotracheal reconstruction procedures undertaken at a pediatric otolaryngology facility. The author found that the procedure does not negatively affect laryngeal growth while providing good results in eliminating subglottic stenosis [20]. Additionally, a 2009 study explored laryngotracheoplasty as an alternative treatment to tracheotomy in infants younger than 6 months old. The authors concluded that the laryngotracheoplasty should be considered as a valid first line treatment to tracheotomy when subglottic stenosis is the primary airway lesion especially as laryngotracheoplasty can be performed as a single-stage procedure [21]. Early treatment may increase quality of life. This work has been reported in line with the SCARE criteria [22].

## 2 Methods

A PubMed literature search with keywords “pediatric subglottic stenosis”, “laryngotracheoplasty”, “laryngotracheal stenosis”, “laryngotracheal reconstruction”, and “costal cartilage graft” going back to the 1970’s was conducted to review clinical cases involving surgical laryngotracheal reconstruction. After review, the most relevant sources to the case discussed below were selected for inclusion. Twenty publications are referenced in this report.

## 3 Report

The patient is a 3-year-old African-American female born prematurely at 30 weeks gestation via emergency cesarean section complicated by placenta previa, twin gestation and absent end diastolic flow. Birth weight was 1.15 kg. The patient was intubated for 4 days after delivery, was transitioned to CPAP and then room air. She was noted to have significant stridor on extubation, was evaluated by ENT and was found to have laryngomalacia. The patient suffered from necrotizing enterocolitis eight days after birth (later, a laparoscopic G-tube was placed when the patient was approximately 6 months old). The patient underwent supraglottoplasty at 2 and ½ months old on and underwent a bilateral inguinal hernia repair on the same date.

The patient was treated for bacterial pneumonia for 2 weeks and was found to have parainfluenza C. infection at approximately 3 months of age. During this period the patient had an abnormal electroencephalogram (EEG) and had an episode of cardiac arrest due to respiratory disorder. The patient failed two post-pneumonia extubation trials at 3 months old and at 3 months and 2 days was recommended for a tracheostomy procedure. The patient’s parents requested a second opinion and the patient was then transferred to the PICU department at Joe DiMaggio Children’s Hospital. There she was evaluated by direct laryngobrochoscopy and was found to have bowing of the vocal cords with subglottic stenosis. The patient received a tracheostomy at 4 months of age due to worsening respiratory status and severe tracheomalacia.

At 6 and ½ months of age, the patient was coming home from a doctor’s appointment when her oxygen tank ran out of oxygen. The patient became progressively hypoxemic in the vehicle with oxygen saturations declining to approximately 60%. The patient was driven directly to Northshore Emergency Department where she was found to be unresponsive, apneic and pulseless. Cardiopulmonary resuscitation was performed, and one dose of epinephrine was given which resulted in the return of spontaneous circulation. The patient was transferred to Joe DiMaggio Children’s Hospital for further evaluation where she was found to be at her neurologic baseline. Thereafter, the patient became a resident of Kidz Korner for consistent nursing care.

At 1 year and 2 months of age, the patient was evaluated by Dr. Ostrower at Joe DiMaggio Children’s Hospital and was found to have severe posterior wall tracheomalacia and left mainstem bronchomalacia. At 1 year and 3 months of age, the patient was found to have mild concentric subglottic stenosis occupying 25% (grade I) of her airway.

At 1 year and 8 months of age, the patient underwent direct microlaryngoscopy, rigid bronchoscopy and balloon dilation of her subglottic stenosis. The patient was found to have bilateral vocal cord edema, grade III subglottic stenosis and proximal tracheomalacia with suprastomal collapse.

**Fig. A2:**
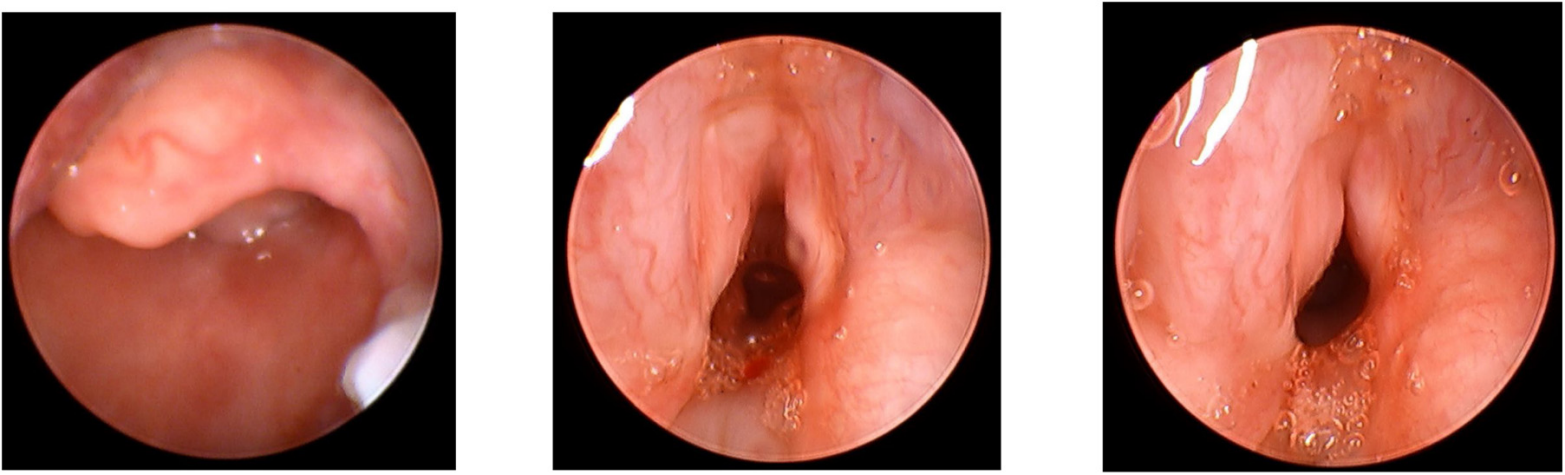
Images - Direct Microlaryngoscopy Showing Grade III Subglottic Stenosis.

At 1 year and 11 months of age, the patient had repeated direct microlaryngoscopy with balloon dilation of her subglottic stenosis. Additionally, the patient underwent CO2 laser ablation and intracapsular tonsillectomy and adenoidectomy. The patient was found to have grade III subglottic stenosis, normal proximal, mid and distal trachea with a large suprastomal granuloma (Fig. A2). Her right and left mainstem bronchi and segmental bronchi had moderate bronchomalacia. Pre-procedure the patient had severe adenoid hypertrophy and with bilateral +4 tonsils.

**Fig. A3:**
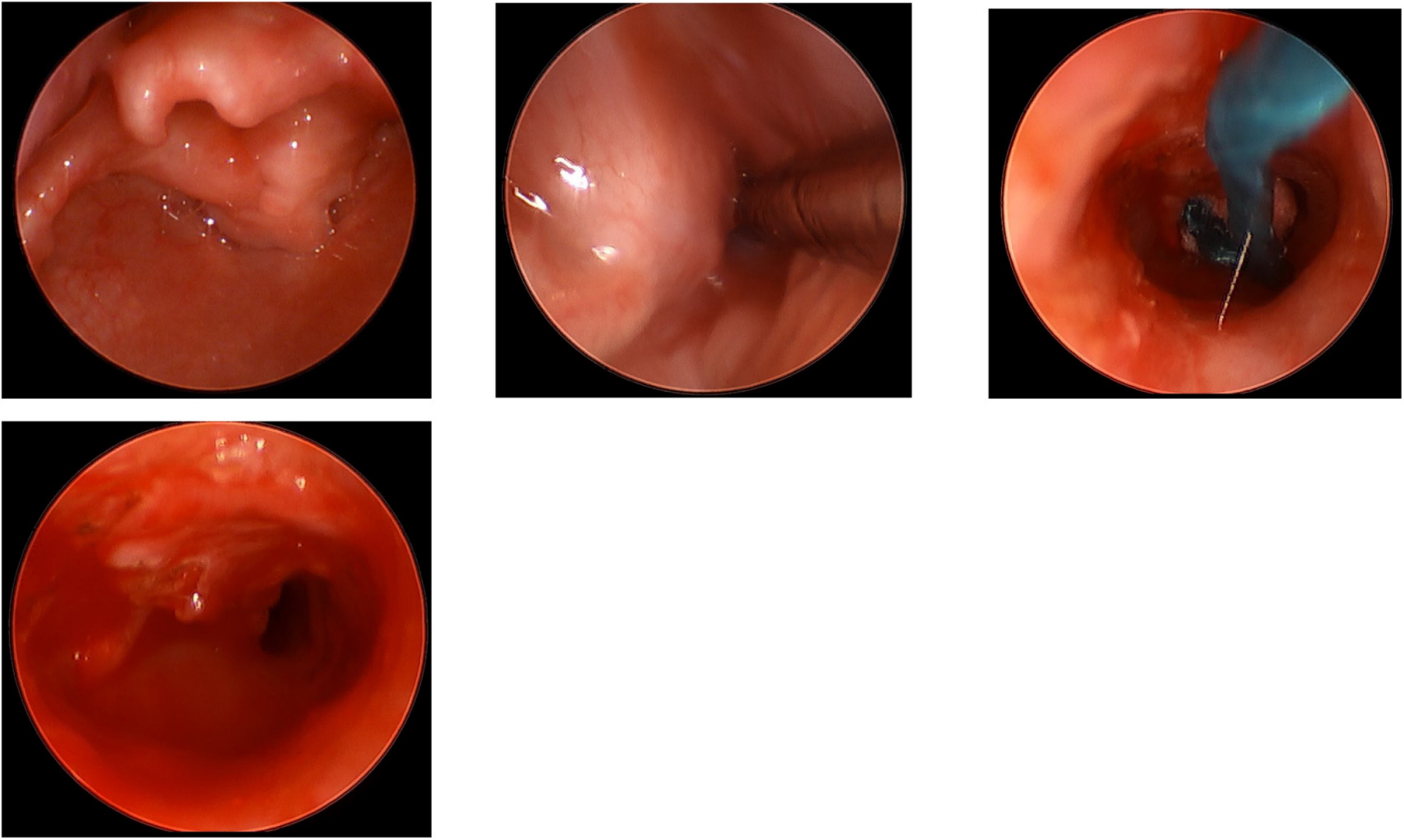
Images – Direct Microlayngoscopy and Rigid Bronchoscopy Showing Grade III Subglottic Stenosis with Suprastomal Granulation in the Proximal Trachea.

At 2 years and 3 months of age, still with grade III subglottic stenosis, the patient had repeat direct microlayngoscopy, rigid bronchoscopy, CO2 laser ablation and balloon dilation. The patient was found to have suprastomal granulation in the proximal trachea (Fig. A3).

**Fig. A4:**
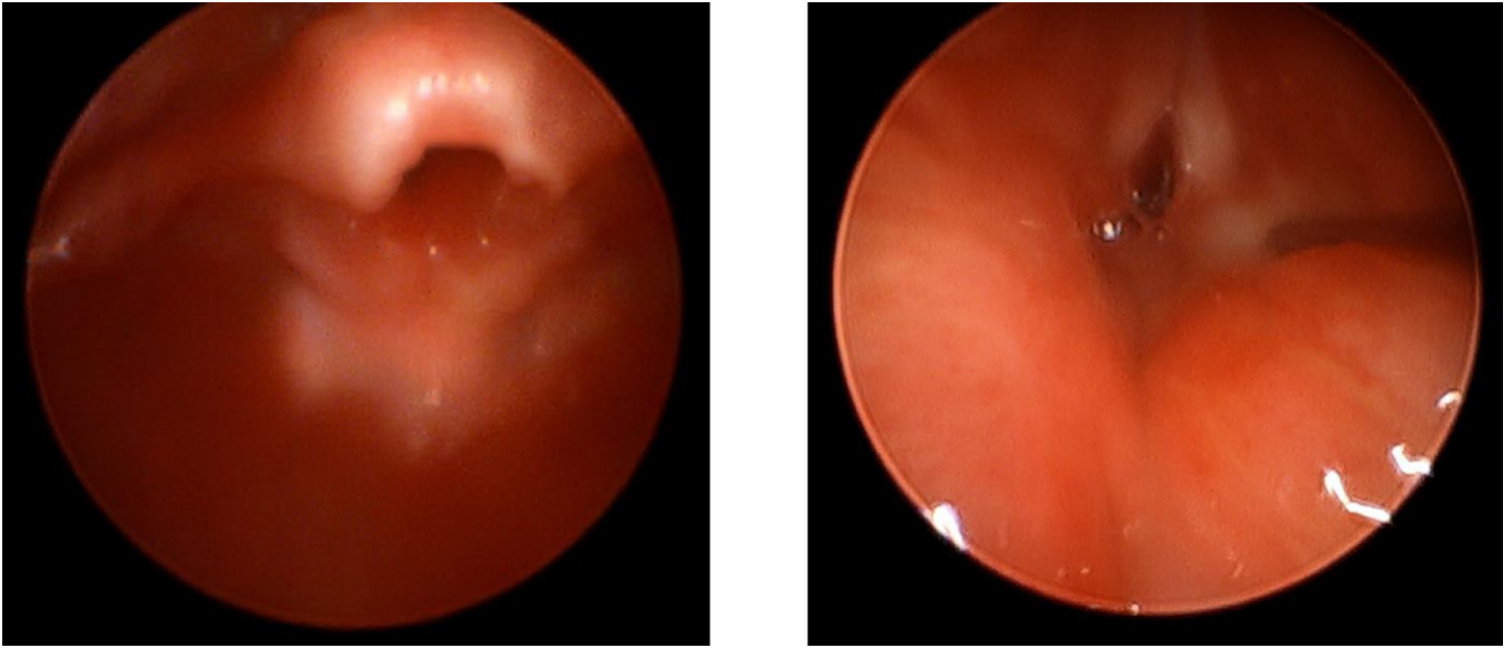
Images- Direct Microlayngoscopy and Rigid Bronchoscopy Showing the Subglottis with 99% Soft, Circumferential Stenosis with Moderate Proximal/Suprastomal Collapse.

At 3 years and 1 month old, for the fourth time, the patient again underwent direct microlayngoscopy and rigid bronchoscopy to follow up on her grade III subglottic stenosis. At this visit, the patient was found to have reduced passive vocal cord mobility (right greater than left), and the subglottis was found to have 99% soft, circumferential stenosis only accepting a 1.9 mm rigid telescope. There was moderate proximal/suprastomal granulation/collapse (Fig. A4). The patient was also evaluated by gastroenterology and had an endoscopy that ruled out gastroesophageal reflux and eosinophilic esophagitis. The patient had been on flovent and omeprazole prophylactically.

**Fig. A5:**
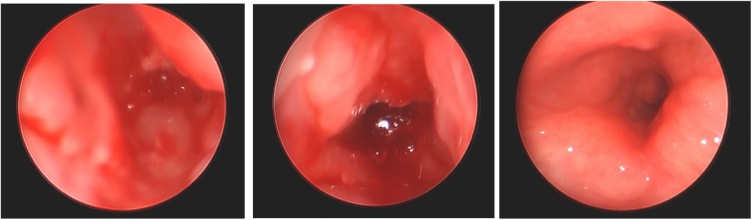
Images – Flexible Laryngoscopy with Visualization of the Nasopharynx and Oropharynx.

At 3 years and 7 months of age, the patient reported to the office for follow up for her severe grade III subglottic stenosis and for pre-op for airway reconstruction. The patient was found to have contracted Coxsackievirus virus (hand, foot, and mouth disease) the month prior, but it was resolved. A pre-operative laryngoscopy was performed showing visualization of the nasopharynx and oropharynx (Fig. A5). Of note, the patient was agitated during the procedure and the supraglottis, glottis and subglottis were not able to be visualized.

**Fig. A6:**
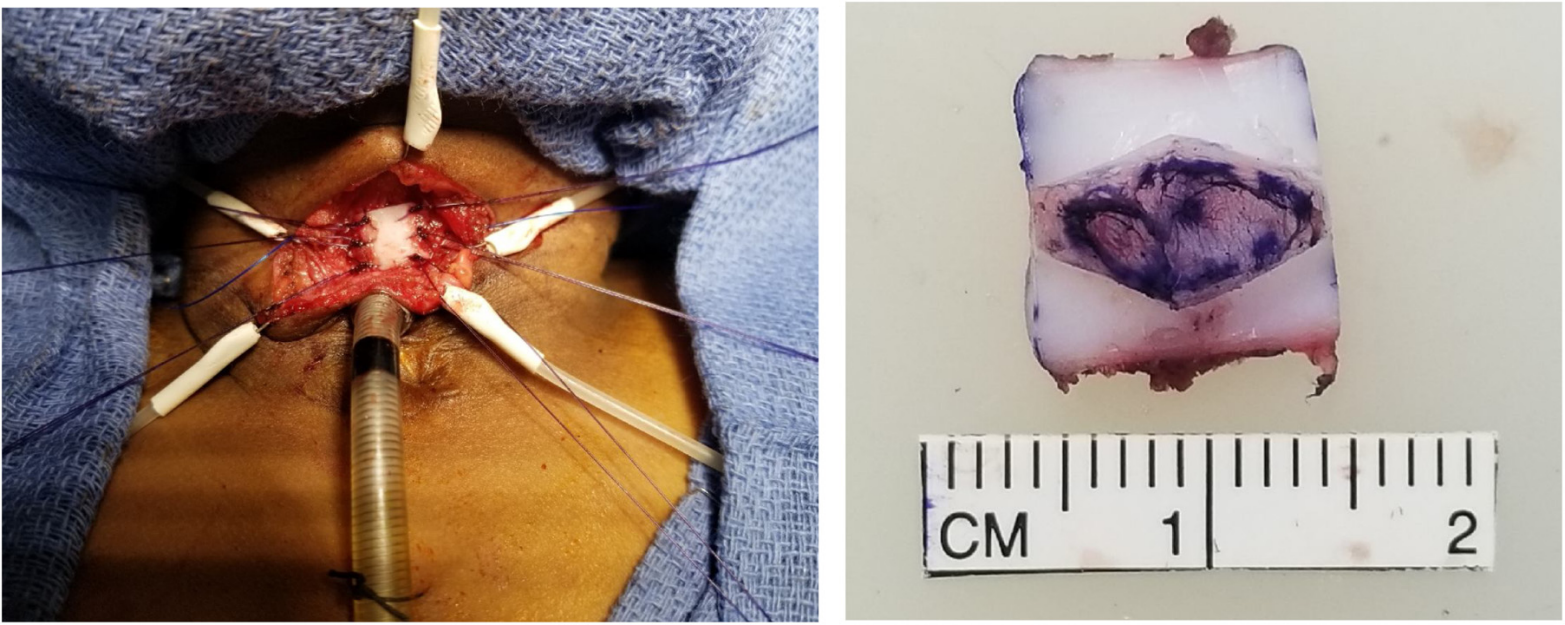
Images - A Double-Stage Laryngotracheoplasty with Anterior/Posterior Costal Cartilage Graft and Stent Placement (Anterior Graft shown).

At 3 years and 8 months of age, Dr. Ostrower with co-surgeon Dr. Brietzke performed a double stage laryngotracheoplasty with anterior/posterior costal cartilage graft and stent placement (Fig. A6). The cartilage graft was harvested from the 8th rib on the right side by Dr. Shah and Dr. Brietzke and measured a total of 16 mm in length. A 4 cm skin incision was made over the patient’s 8th rib on the right side with a #15 blade. Dissection was carried through the subcutaneous tissue and muscle with bipolar cautery. The bony-cartilaginous junction of the 8th right rib was identified using a sharp 25-gauge needle that pierced the rib laterally to delineate bone from cartilage. The rib was divided sharply with a # 15 blade and was lifted superiorly. The medial edge was identified, the pleura of the medial edge was protected, and a # 15 blade was used to remove some of the costal cartilage while keeping the anterior perichondrium intact. The resulting rib graft was placed in sterile saline on a back table. A Valsalva maneuver was performed with 30 cm of water showing no air leak and confirming that the pleura had not been violated. The muscle layer was closed with 3-0 Vicryl in a horizontal mattress stich pattern and the skin was closed with 3-0 Vicryl and 4-0 Monocryl with Dermabond.

**Fig. A7:**
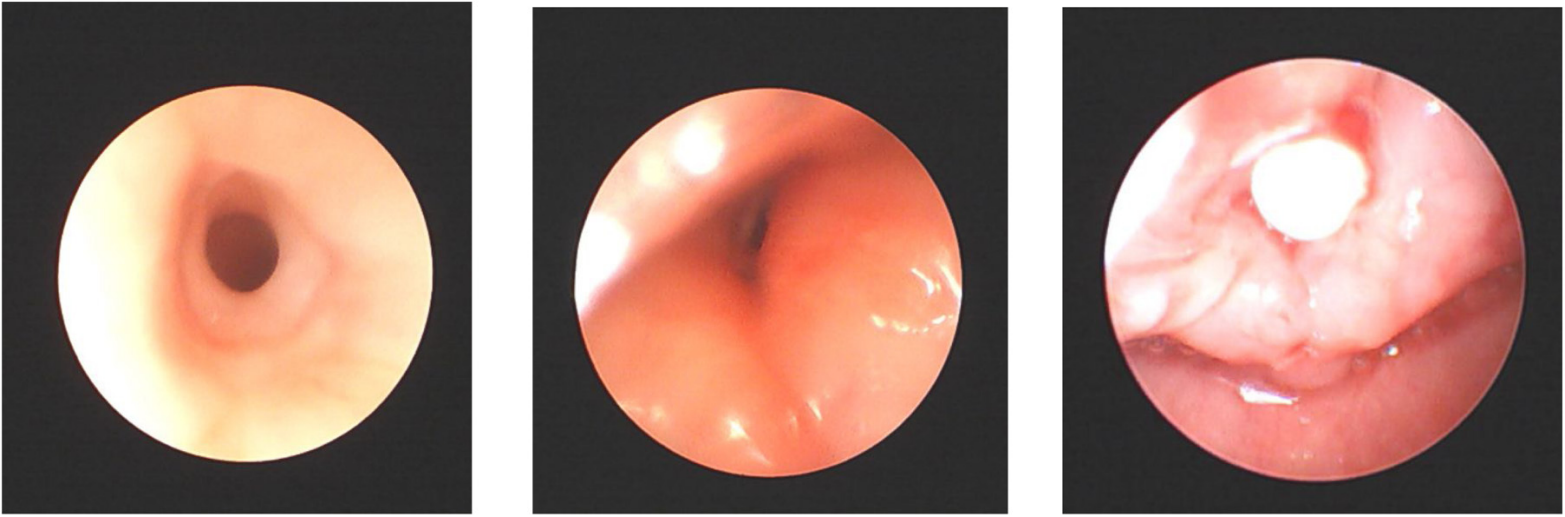
Images – Tracheal Bronchoscopy.

Next, an approximate 4 cm horizontal neck incision was performed that was spaced above the existing tracheostomy stoma. Subplatysmal flaps were elevated superiorly and inferiorly with care not to enter the tracheostomy stoma. Strap muscles were identified and retracted laterally exposing the airway. The thyroid cartilage, thyroid notch, cricothyroid membrane, cricoid cartilage, and first tracheal ring were positively identified. There was significant visible external scarring surrounding the cricoid. A needle was placed through the tracheal cartilage with concurrent direct endoscopy and bronchoscopy (Fig. A7) to verify the incision at the midline subglottic area. A #11 blade was used to perform midline incision in the cricoid and trachea exposing the area of subglottic stenosis. Care was taken not to enter the area of the tracheotomy stoma. Dr. Ostrower then performed a midline incision of the posterior trachea and posterior cricoid cartilage. Careful measurements were taken and the anterior and posterior cartilage grafts were carved on a back table.

The posterior graft was carved into the classic “T” shape at 5 mm in length × 3 mm perichondral width with 2 mm flanges bilaterally. The anterior graft was carved to a length of 11 mm with elliptical perichondral surface of 5 mm in greatest width with 3 mm depth and generous flanges. The posterior cartilage graft was placed by distracting the cricoid ring. The flange of the posterior cartilage graft and the natural recoil of the cricoid ring secured the graft into place. The airway was examined by telescope to confirm proper graft placement.

A 6 mm Rutter stent was then placed in the airway with a cap on the superior end. Placement was verified by direct laryngoscopy and bronchoscopy. The stent was secured with a single Prolene suture pass through the left tracheal sidewall, the stent itself, and the right tracheal sidewall. The anterior costochondral graft was secured using 6 Moncryl sutures with 3 sutures on the left and 3 sutures on the right. The Monocryl sutures did not enter the airway. Irrigation was placed with no evidence of air leak. The free ends of the Prolene suture were tied over a section of 18-gauge Angiocath tubing and left below the skin anterior to the tracheal for location during later stent removal.

The wound was closed in layers; first with closure of the strap muscles, then subcutaneous tissue closure, the subcuticular tissue closure. Steri-Strips with Mastisol were placed on the skin. An armored endotracheal tube was removed and a flexed Bivona cuffed trach was replaced and secured with Velcro necktie. The patient was awakened and transferred to the PICU for overnight observation.

The patient remained in the PICU for 5 days. On day 3 Robinul was initiated due to copious secretion production and tracheostomy tube requiring frequent suctioning. On postoperative day 4, the patient’s sputum culture revealed *P*. *aeruginosa* and the patient was started on ciprofloxacin by G-tube, levofloxacin and ciprodex treatments by tracheostomy tube. Patient was afebrile for over 48 h and discharged on post-operative day 6.

**Fig. A8:**
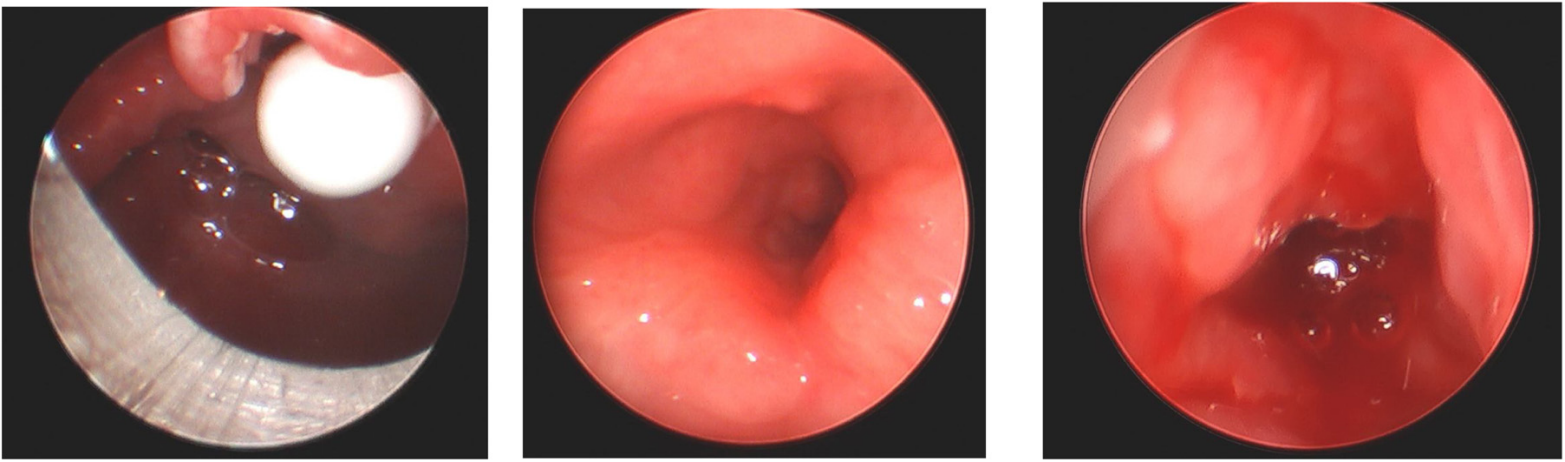
Images - Direct Microlaryngoscopy and Rigid Bronchoscopy.

**Fig. A9:**
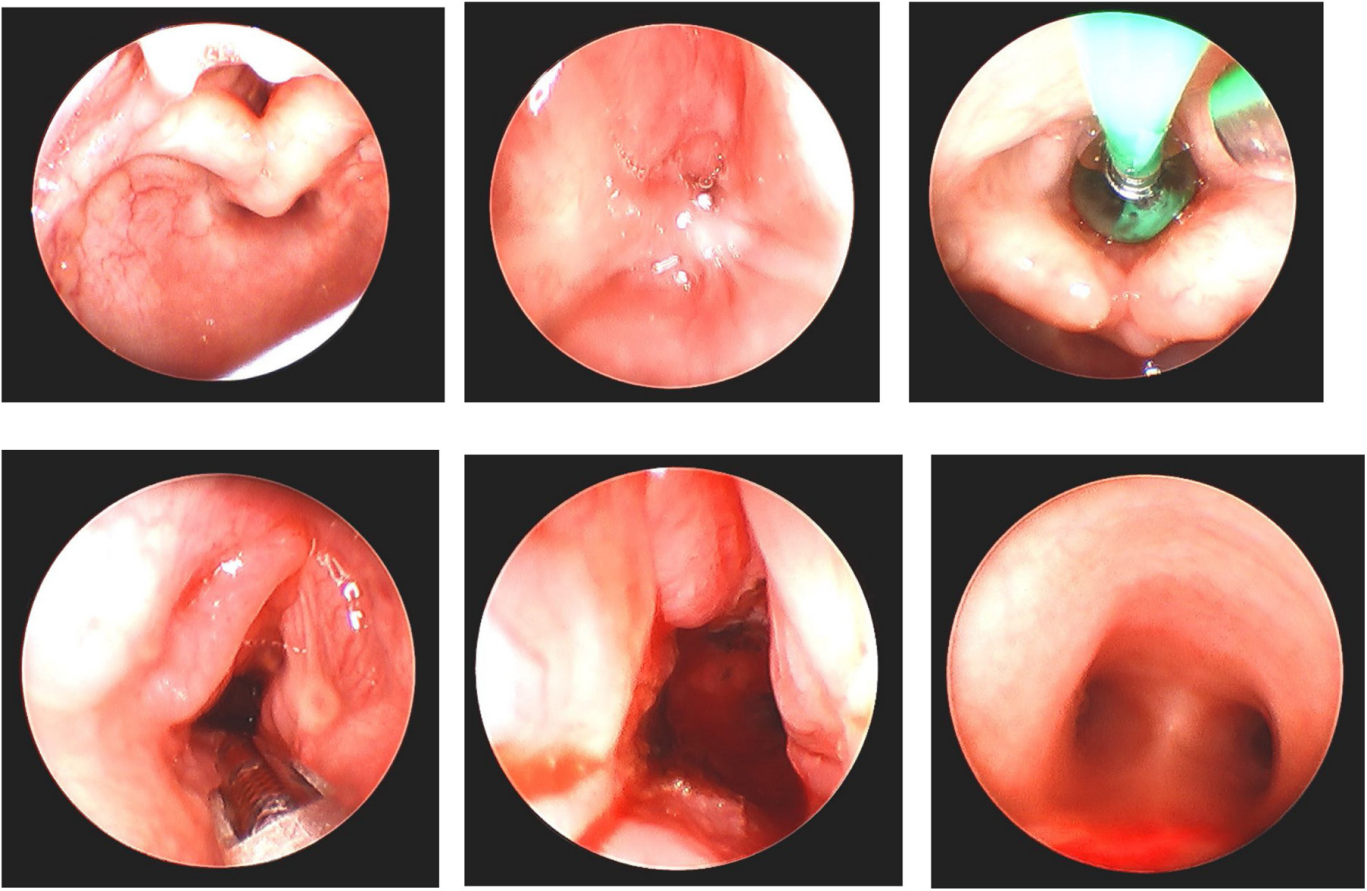
Images - Follow-up Microlaryngoscopy and Bronchoscopy with. CO2 Laser Ablation and Balloon Dilation.

At 3 years and 9 months of age, the patient underwent removal of the laryngeal stent, direct microlaryngoscopy and rigid bronchoscopy. Visualization showed a patent subglottis with bilateral granulation tissue with graft in place. The patient had normal proximal, mid and distal tracheal with normal right and left mainstem bronchi to the segmental bronchi (Fig. A8). One month later, the patient returned for follow-up repeat microlaryngoscopy and bronchoscopy that showed some scaring and so the patient underwent CO2 laser ablation and balloon dilation (Fig. A9).

As of March 2019, the patient continues to have regular follow up laryngoscopy/bronchoscopy with dilation. Ultimately the goal for the patient, once the laryngotracheal reconstruction has healed, is to be able to maintain her own airway and no longer be tracheostomy tube dependent.

## 4 Conclusion

The case described above demonstrates the appropriateness of the double-stage laryngotracheal reconstruction procedure with cartilage graft and stent placement for the surgical treatment of grade III subglottic stenosis in the pediatric patient. The case also demonstrates the correct prophylactic use of omeprazole (proton pump inhibitor) in a patient with subglottic stenosis and it illustrated the correct treatment of post-laryngotracheal reconstruction P. *aeruginosa* stent colonization without complications.

One month after laryngotracheoplasty the operative stent was successfully removed. Four weeks after stent removal the patient will return for follow-up repeat direct laryngoscopy and bronchoscopy with the anticipation that the tracheostomy tube will be removed and the patient will maintain her own airway. Tracheostomy tube removal may be slightly delayed due to the repeated CO2 laser ablation and balloon dilation adjuvant treatment. If the stenotic segment has again formed extensive granulation tissue, a repeat CO2 laser ablation with balloon dilation adjuvant treatment will be conducted. The patient will be seen for follow up every two weeks until tracheostomy tube removal is accomplished.

The most important aspect of this case is its demonstration that the double-stage laryngotracheoplasty allows for the surgical correction of more advanced grades of subglottic stenosis (grade III and grade IV). Before this advance in surgical technique, the balloon dilation (with or without adjuvant CO2 laser ablation) treatment modality could only successfully manage lesser grade (grade I and grade II) subglottic stenosis.

This case is in line with the cited literature and is an example of successful otolaryngologic treatment of rare condition due to neonatology advances in airway management away from intubation and toward noninvasive positive pressure airway support. Although becoming less often necessary, both the single-stage and double-stage laryngotracheoplasty with cartilage graft are useful skills and remain the best modality to successfully treat the pediatric patient with advanced grade (grade III or grade IV) subglottic stenosis.

## Conflicts of interest

The authors acknowledge no conflicts of interest.

## Sources of funding

No funding was provided for this research.

## Ethical approval

The case report is exempt form ethical approval.

## Consent

As we were unable to get patient consent we have a general release from Joe DiMaggio Children's Hospital stating that they allow the case to be published. Dr. Ostrower is the Medical Director of Pediatric Otolaryngology at Joe DiMaggio Children's Hospital in Hollywood, FL where this case took place. The Editor in Chief and has approved this and the document is available to review at the journal’s request.

## Author contribution

Nathan Montoya Albrecht, MPH, OMS IV, Edward Via College of Osteopathic Medicine – Auburn, AL – Conceptualized, collected data, created the formal analysis, investigation, methodology, administered the project, primary writer of the original and all subsequent drafts.

Samuel Ostrower, MD, Medical Director, Pediatric Otolaryngology Joe DiMaggio Children’s Hospital - Hollywood, FL - conceptualized, curated data, primary reviewer and editor of the original and all subsequent drafts.

## Registration of research studies

researchregistry4692.

## Guarantor

Nathan Albrecht

## Provenance and peer review

Not commissioned, externally peer-reviewed.
